# Predictors of mortality within the first year post-hepatectomy for hepatocellular carcinoma

**DOI:** 10.1186/s43046-022-00113-8

**Published:** 2022-04-04

**Authors:** Sanaa Sheriff, Sudharsan Madhavan, Geraldine Yanlei Lei, Yiong Huak Chan, Sameer P. Junnarkar, Cheong Wei Huey, Jee Keem Low, Vishal G. Shelat

**Affiliations:** 1 Ministry of Health Holdings, Singapore, Singapore; 2 Biostatistics Unit, National University Health System, Singapore, Singapore; 3 Hepato-Pancreatico-Biliary Surgery, Department of General Surgery, Tan Tock Seng Hospital, Singapore, Singapore

**Keywords:** Liver, Surgery, Hepatectomy, Hepatocellular carcinoma, Mortality

## Abstract

**Background:**

Hepatic resection (HR) for hepatocellular carcinoma (HCC) is safe with good perioperative and long-term oncologic outcomes. There is a paucity of data with regards to intermediate-term outcomes (i.e., beyond 90-day and within 1-year mortality). This paper studies the risk factors for within 1-year mortality after elective HR with curative intent in patients with HCC.

**Methods:**

An audit of patients who underwent curative HR for HCC from January 2007 to April 2016 was conducted. Univariate and multivariate analysis were sequentially performed on perioperative variables using Cox-regression analysis to identify factors predicting intermediate-term outcomes defined as within 1-year mortality. Kaplan–Meier survival curves and hazard ratios were obtained.

**Results:**

Three hundred forty-eight patients underwent HR during the study period and 163 patients had curative hepatectomy for HCC. Fifteen patients (9.2%) died within 1-year after HR. Multivariate analysis identified Child-Pugh class B/C (HR 5.5, *p* = 0.035), multinodularity (HR 7.1, *p* = 0.001), macrovascular invasion (HR 4.2, *p* = 0.04) postoperative acute renal failure (HR 5.8, *p* = 0.049) and posthepatic liver failure (HR 9.6, *p* = 0.009) as significant predictors of 1-year mortality.

**Conclusion:**

One-year mortality following HR for HCC remains high and can be predicted preoperatively by multinodularity, Child-Pugh class, and macrovascular invasion. Postoperative acute renal failure and liver failure are associated with 1-year mortality.

## Background

Hepatocellular carcinoma (HCC) is the fifth most prevalent cancer worldwide and the third most frequent cause of cancer-related death [1]. Locally, HCC is the third and fourth most frequent cause of cancer-related death among men and women, respectively [2]. Due to the shortage of donor livers for liver transplant (LT), hepatic resection (HR) is the first-line curative option and has low morbidity and mortality. However, recurrence rates remain high with an impact on delayed mortality [3, 4]. Tumor size and multinodularity are known predictors of early recurrence [3]. Many units, including ours, have witnessed that 90-day mortality is significantly higher than 30-day mortality [4], leading to underestimation of perioperative death when using the traditional 30-day mortality as a key performance indicator of HR outcomes. Short-term perioperative outcomes report events up to 90-days. Long-term oncologic outcomes universally report 1-year, 3-year, or 5-year disease-free survival (DFS) or overall survival (OS). Thus, there is a hiatus in surgical oncology for reporting intermediate-term outcomes after 90 days and before 12 months. In colon cancer patients, reports found underestimation of 1-year mortality and identified postoperative complications, emergency resection, and comorbidities as risk factors [5]. Age, future liver remnant (FLR), and hepatic insufficiency [6] are associated with 90-day mortality risk following HR. Beyond 90-day mortality in HR is poorly studied and reported. In addition to tumor biology, early recurrence or treatment-related complications impact beyond 90-day mortality outcomes. Identification of mortality predictors within the first year post-hepatectomy would aid in clinical decision-making, in particular when considering less invasive treatment options and alternative strategies to HR with lower morbidity risk, as well as in patient enrolment into clinical trials.

Radiofrequency ablation (RFA) with trans-arterial chemoembolization (TACE) has been studied and reported to have equivalent oncological outcomes compared with HR [7, 8]. A propensity score-matched analysis of 154 HCC patients with single 2–3-cm tumors by Lee et al. [9] reported no noticeable difference in local tumor progression, intrahepatic distant recurrence, disease-free survival, and overall survival when comparing HR with TACE–RFA combination therapy. A recent meta-analysis by Giu et al. [7] also concludes that TACE–RFA combination therapy is associated with lower morbidity compared with surgery with comparable oncologic outcomes. Hence, in addition to 90-day mortality risk predictors, knowledge of predictors within 1-year mortality is vital as this could impact treatment recommendation, patients’ quality of life, and patient choice. Our study aims to identify all factors predicting mortality within the first year for patients undergoing HR with curative intent for HCC.

## Methods

This is a retrospective audit of patients operated for HR at a university-affiliated hospital from January 2007 to April 2016. Survival data of all the patients was obtained prospectively until February 2018. Patients included in the study were those who underwent curative open or laparoscopic surgery for HCC. Patients with cholangiocarcinoma and metastatic liver nodules were excluded. All the patients were discussed at the multidisciplinary tumor board meeting prior to surgery.

An anesthetist assessed every patient preoperatively at the pre-admissions counseling and evaluation clinic. Nonsurgical treatment modalities were offered for patients deemed unfit by the operating surgeon or those with a high cardiovascular risk as adjudged by an anaerobic threshold of < 11 ml/min/kg. An ICG retention value of > 15% at 15 min precludes major HR. Our institute offers liver-directed chemo- and radio-embolization as well as local thermal ablative therapies for suitable patients. The HPB consensus guidelines were used to recognize those with satisfactory FLR [10]. The Brisbane 2000 classification [11] was used to define the types of resection. Only minor HRs were offered to Child-Pugh class B/C patients. The choice of technique (i.e., open versus laparoscopic or anatomical versus nonanatomical resection) was left to the individual surgeon.

Intraoperative findings and postoperative complications were recorded. R1 resection indicates a histologically positive margin with tumor cells within 1 mm from the margin. The presence of any combined HCC and cholangiocarcinoma cases (*n* = 2) were excluded. Post-hepatectomy liver failure (PHLF) 1 (50–50 criteria), PHLF2 (peak serum bilirubin criteria), and PHLF3 (ISGLS criteria) were utilized as per their original definitions [12–14]. Length of stay was calculated from the date of surgery to discharge date, both dates inclusive. Following discharge, patients were followed up with a physical examination, serum liver function tests, alpha-fetoprotein (AFP), and computed tomography (CT) scan at regular intervals of 3 to 4 months for the first year and 4 to 6 months thereafter. Within 1-year mortality is defined as all-cause mortality within 12 months of the date of HR.

Descriptive statistics for quantitative data were expressed as median and range, while the qualitative data was expressed as *n* (%). For nominal data, chi-square or Fisher’s exact test was performed. Paired metric data were analyzed using the Wilcoxon signed-rank test. Variables with *p* values < 0.1 in univariate analysis were entered for multivariate analysis with the Cox proportional hazard model. Statistical significance was defined as *p* < 0.05. Kaplan–Meier survival curves were generated for the pre-, intra-, and postoperative independent risk factors from multivariate analysis. All analyses were done using IBM SPSS Statistical Analysis Version 20.0.

## Results

Between January 2007 and April 2016, 400 patients underwent HR at our institution. Thirty patients (7.5%) were excluded due to missing data for perioperative variables, and twenty-two patients (5.5%) were excluded for missing long-term oncologic outcome data. Out of 348 patients, 163 patients (46.8%) with a median age of 66 years (37–87 years) underwent HR with curative intent, and the diagnosis of HCC was confirmed histologically. The baseline characteristics of the patients are summarized in Table 1. The median tumor diameter was 40 mm (1–200 mm), with 68 patients (41.7%) having tumors ≥ 50 mm. Seventeen (10.4%) were of Child-Pugh class B/C, and over half (51.5%) had Hepatitis B. Seventy-four patients (45.4%) underwent major HR, and 101 (62.0%) had laparoscopic hepatectomy (Table 1). Thirty-day mortality was 3.7% (*n* = 6), and 90-day mortality was 4.9% (*n* = 8). Fifteen patients (9.2%) died within the first year post-hepatectomy (Table 2). The median survival of patients with 1-year mortality was 9 months (range 0–11 months).

**Table 1:** Demographic and clinic profile

	Number (***n*** = 163)
Age in years (*median*) (range)	66 (37 – 87)
Male	135 (83%)
American Society of Anesthesiology score > 2	89 (54.6%)
Hypertension	108 (66.3%)
Hyperlipidemia	90 (55.2%)
Diabetes mellitus	69 (42.3%)
Ischemic heart disease	26 (16.0%)
Body mass index > 27	24 (14.7%)
Hepatitis B	84 (51.5%)
Hepatitis C	18 (11%)
Child-Pugh class B/C^a^	17 (10.4%)
Alpha-fetoprotein level ≥ 1000	26 (16%)
Hemoglobin < 10 g/dL	5 (3.1%)
Albumin (mg/dL) (*mean*)	37.60
Creatinine (μmol/L) (*mean*)	87.69
Bilirubin (μmol/L) (*mean*)	17.47
Indocyanine green retention time at 15 min (*mean*)	9.01

^a^One patient was Child-Pugh class C.

**Table 2:** Perioperative and oncologic outcomes

	Number (***n*** = 163)
Tumor size (mm), median (range)	40 (1 – 200)
Macrovascular invasion	18 (11%)
Microvascular invasion	47 (28.8%)
Pringles maneuver used	27 (16.6%)
Competing interests	Competing interests
Major resection	74 (45.4%)
Right hemihepatectomy	37 (50%)
Left hemihepatectomy	21 (28.4%)
Right trisectionectomy	12 (16.2%)
Left trisectionectomy	1 (1.4%)
Central hepatectomy	3 (4%)
Minor resection	89 (54.6%)
Laparoscopic resection	101 (62%)
Multifocal (≥ 2) tumors	43 (26.4%)
Giant (≥ 10 cm) tumor	32 (19.6%)
Blood transfusion	30 (18.4%)
Pneumonia	14 (8.6%)
Acute renal failure	7 (4.3%)
Urinary tract infection	6 (3.7%)
Pulmonary embolism	1 (0.6%)
Intra-abdominal abscess	4 (2.5%)
Bile leak	3 (1.8%)
Superficial surgical site infection	7 (4.3%)
Post-hepatectomy liver failure 1 (50–50 criteria)	6 (3.7%)
Post-hepatectomy liver failure 2 (peak serum bilirubin criteria)	7 (4.3%)
Post-hepatectomy liver failure 3 (ISGLS criteria)	25 (15.3%)
30-Day mortality	6 (3.7%)
90-Day mortality	8 (4.9%)
One-year mortality	15 (9.2%)

*ISGLS* International Study Group on Liver Surgery

Table 3 summarizes the univariate and multivariate analysis of preoperative variables. The following factors were associated with 1-year mortality: albumin < 35 g/dL (*p* = 0.064), prognostic nutritional index (*PNI*) < 45 (*p* = 0.021), platelet–lymphocyte ratio (*PLR*) ≥ 150 (*p* = 0.089), neutrophil–lymphocyte ratio (*NLR*) ≥ 5 (*p* = 0.038) and Child-Pugh B/C (*p* = 0.004). Multivariate analysis identified Child-Pugh B or C (HR 5.5, *p* = 0.035, 95% confidence interval (*CI*) 1.130–26.551), and the presence of two or more tumors (HR 7.1, *p* = 0.001, 95% *CI* 2.305–21.899) as preoperative factors that were independently associated with mortality.

**Table 3:** Univariate and Multivariate analysis of preoperative variables predicting one-year mortality

*n* = 163	Univariate analysis		Multivariate analysis	
	*p* value	HR (95% CI)	*p* value	HR (95% CI)
Body mass index > 27	0.297	0.039 (0.000–17.449)		
Age > 60 years	0.133	0.453 (0.161–1.272)		
Sex	0.257	0.037 (0.000–11.176)		
Hepatitis B	0.671	0.803 (0.291–2.213)		
Hepatitis C	0.584	0.568 (0.075–4.317)		
Hemoglobin < 10g/dL	0.402	2.380 (0.313–18.110)		
Platelet < 150 × 10^9^/L	0.986	0.989 (0.279–3.504)		
Albumin < 35 g/L	0.064	2.656 (0.945–7.466)	0.453	0.549 (0.114–2.633)
Prognostic nutritional index < 45	0.021	3.545 (1.211–10.375)	0.103	2.974 (0.802–11.030)
Platelet/lymphocyte ratio ≥ 150	0.089	2.412 (0.875–6.652)	0.189	2.112 (0.692–6.447)
Neutrophil/lymphocyte ratio ≥ 5	0.038	3.368 (1.072–10.585)	0.839	0.855 (0.188–3.881)
Alpha-fetoprotein ≥ 1000 ng/mL	0.244	1.973 (0.628–6.198)		
Tumor (≥ 10 cm)	0.469	1.526 (0.486–4.794)		
Tumor (≥ 5 cm)	0.147	2.149 (0.765–6.037)		
Bilirubin > 31 μmol/L	0.531	0.046 (0.000–710.247)		
***Child-Pugh B/C***	***0.004***	***4.941*** **(*1.685–14.491*)**	***0.035***	***5.479*** **(*1.130–26.551*)**
American Society of Anesthesiology score (ASA) > 2	0.319	1.727 (0.590–5.052)		
Eastern Cooperative Oncology Group (ECOG) > 1	0.726	1.437 (0.189–10.930)		
***Tumors*** **(*≥ 2*)**	***0.001***	***5.897 (2.105–17.262)***	***0.001***	***7.105*** **(*2.305–21.899*)**

*CI* Confidence interval

Table 4 summarizes the univariate and multivariate analysis of intraoperative variables. The following factors were associated with 1-year mortality: open HR (*p* = 0.060), major HR (*p* = 0.068), macrovascular invasion (MAVI) (*p* = 0.001), microvascular invasion (*p* = 0.035), and blood loss of more than 1000 ml (*p* = 0.053). Multivariate analysis identified macrovascular invasion (HR 4.2, *p* = 0.043, 95% *CI* 1.046–17.191) as the only intraoperative factor that was independently associated with 1-year mortality. Eighteen patients (11.0%) were reported to have MAVI intraoperatively. Six patients with MAVI died within 1 year.

**Table 4:** Univariate and multivariate analysis of intraoperative variables predicting 1-year mortality

*n* = 163	Univariate analysis		Multivariate analysis	
	*p* value	HR (95% CI)	*p* value	HR (95% CI)
Access	0.060	0.240 (0.054–1.064)	0.223	0.368 (0.74–1.840)
Open conversion	0.370	0.041 (0.000–43.629)		
Major resection	0.068	2.717 (0.929–7.951)	0.682	1.282 (0.391–4.20)
Tumor rupture	0.677	0.650 (0.085–4.941)		
Pringles	0.269	1.907 (0.607–5.989)		
Tumor necrosis	0.285	1.795 (0.614–5.253)		
***Macrovascular invasion***	***0.001***	***6.102*** **(*2.166–17.187*)**	***0.043***	***4.241*** **(*1.046–17.191*)**
Microvascular invasion	0.035	2.976 (1.079–8.211)	0.864	1.127 (0.286–4.448)
R1 resection	0.836	0.049		
Bile leak	0.145	4.522 (0.594–34.453)		
Blood loss (> 1000 mL)	0.053	2.771 (0.986–7.789)	0.264	1.841 (0.631–5.373)
Blood transfusion	0.849	1.131 (0.319–4.008)		

*CI* Confidence interval

Table 5 summarizes the univariate and multivariate analysis of postoperative variables. Multiple factors were associated with 1-year mortality: intensive care unit admission (*p* < 0.001), pneumonia (*p* < 0. 001), acute renal failure (ARF) (*p* < 0. 001), intra-abdominal abscess (*p* = 0.006), PHLF1 (*p* < 0.001), PHLF2 (*p* < 0.001), and PHLF3 (*p* < 0.001). Multivariate analysis identified ARF (HR 5.8, *p* = 0.049, 95% *CI* 1.009–33.294) and PHLF3 (HR 9.6, *p* = 0.009, 95% *CI* 1.778–51.320) to be independently associated with 1-year mortality. Twenty-eight patients (17.1%) experienced PHLF3, out of which almost 40% died within 1 year. There was a significantly higher incidence of PHLF3 in patients experiencing one-year mortality (73.3% vs. 9.5%). Similarly, a total of seven patients experienced ARF, and six died within 1 year.

**Table 5:** Univariate and multivariate analysis of postoperative variables predicting one-year mortality

*n* = 163	Univariate analysis		Multivariate analysis	
	*p* value	HR (95% CI)	*p* value	HR (95% CI)
Pneumonia	0.001	16.233 (5.830–45.201)	0.193	2.856 (0.588–13.875)
Cardiac event	0.001	11.241 (3.961–31.901)	0.308	2.603 (0.414–16.381)
Ascites	0.023	4.325 (1.218–15.358)	0.448	2.174 (0.292–16.187)
Pleural effusion	0.001	5.898 (2.136–16.286)	0.709	1.365 (0.266–7.002)
***Acute renal failure***	***0.001***	***27.455*** **(*9.329–80.802*)**	***0.049***	***5.795*** **(*1.009–33.294*)**
Urinary tract infection	0.610	0.047 (0.000–5964.790)		
Superficial surgical site infection	0.004	6.437 (1.813–22.857)	0.326	3.172 (0.317–31.719)
Intra-abdominal abscess	0.006	8.286 (1.854–37.041)	0.443	0.433 (0.056–3.535)
Ileus	0.010	4.075 (1.390–11.943)	0.721	1.411 (0.214–9.328)
Post-hepatectomy liver failure 1 (50–50 criteria)	0.001	15.676 (4.839–50.783)	0.524	1.763 (0.309–10.057)
Post-hepatectomy liver failure 2 (peak serum bilirubin criteria)	0.001	11.997 (3.751–38.368)	0.785	0.728 (0.075–7.115)
***Post-hepatectomy liver failure 3 (ISGLS criteria)***	***0.001***	***19.761*** **(*6.251–62.475*)**	***0.009***	***9.553*** **(*1.778–51.320*)**
High dependency stay	0.707	1.475 (0.194–11.220)		
Intensive care unit stay	0.001	9.238 (3.149–27.100)	0.628	1.552 (0.263–9.167)

*CI* Confidence interval

**Fig. 1:**
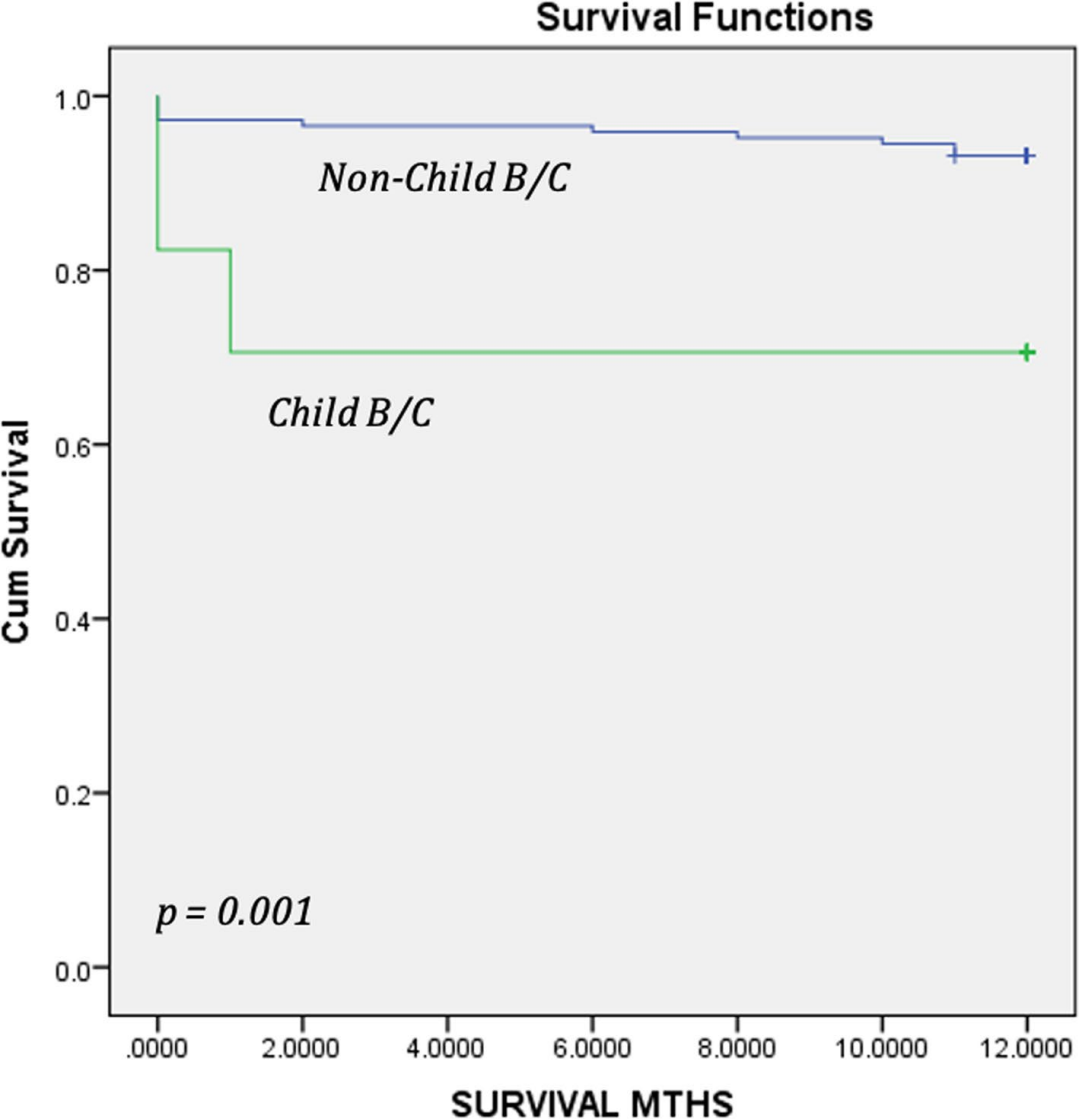
Kaplan–Meier survival curve for 1-year survival of Child-Pugh class B/C, *p* = 0.001

**Fig. 2:**
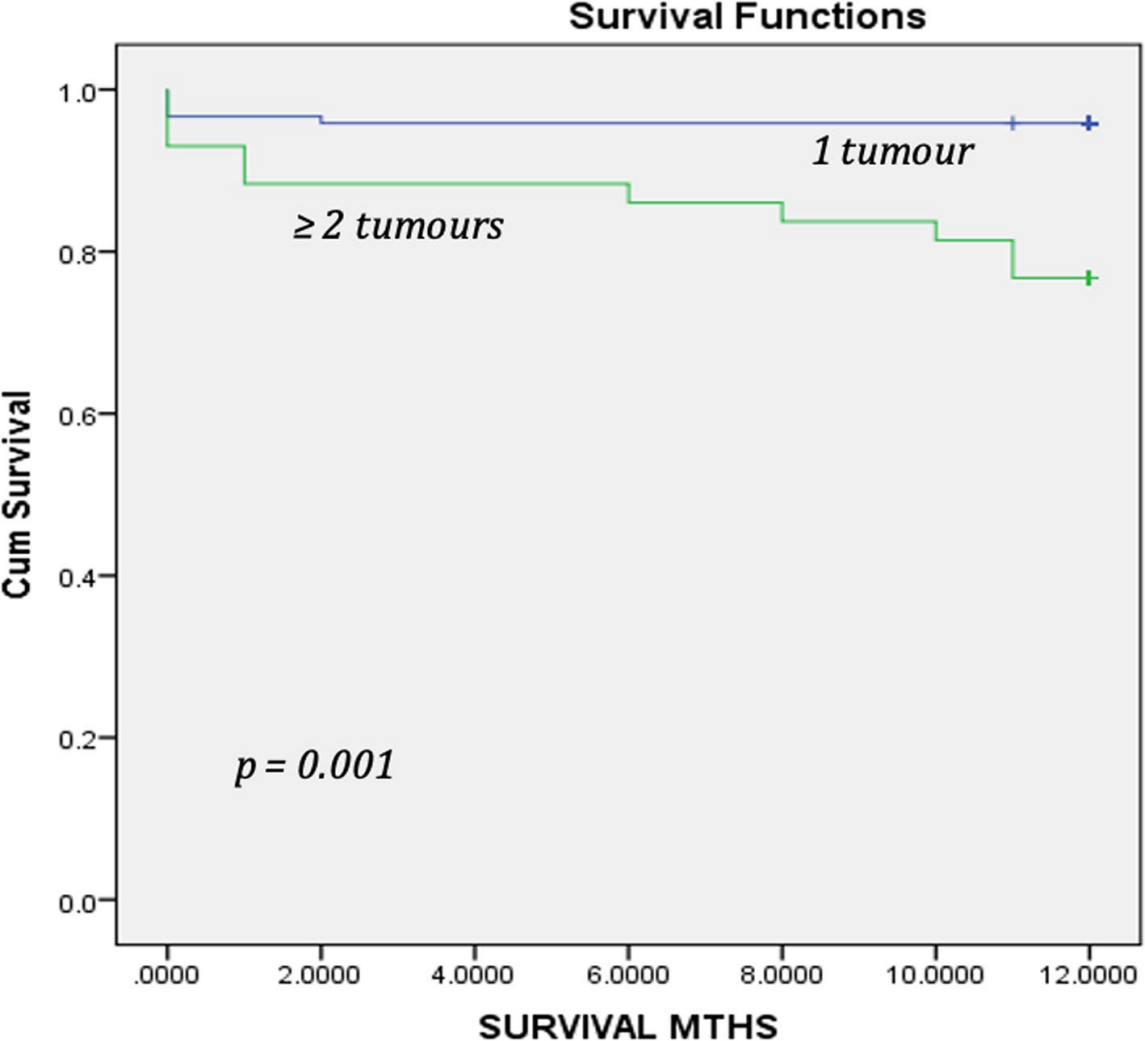
Kaplan–Meier survival curve for 1-year survival of multifocal tumor, *p* = 0.001

**Fig. 3:**
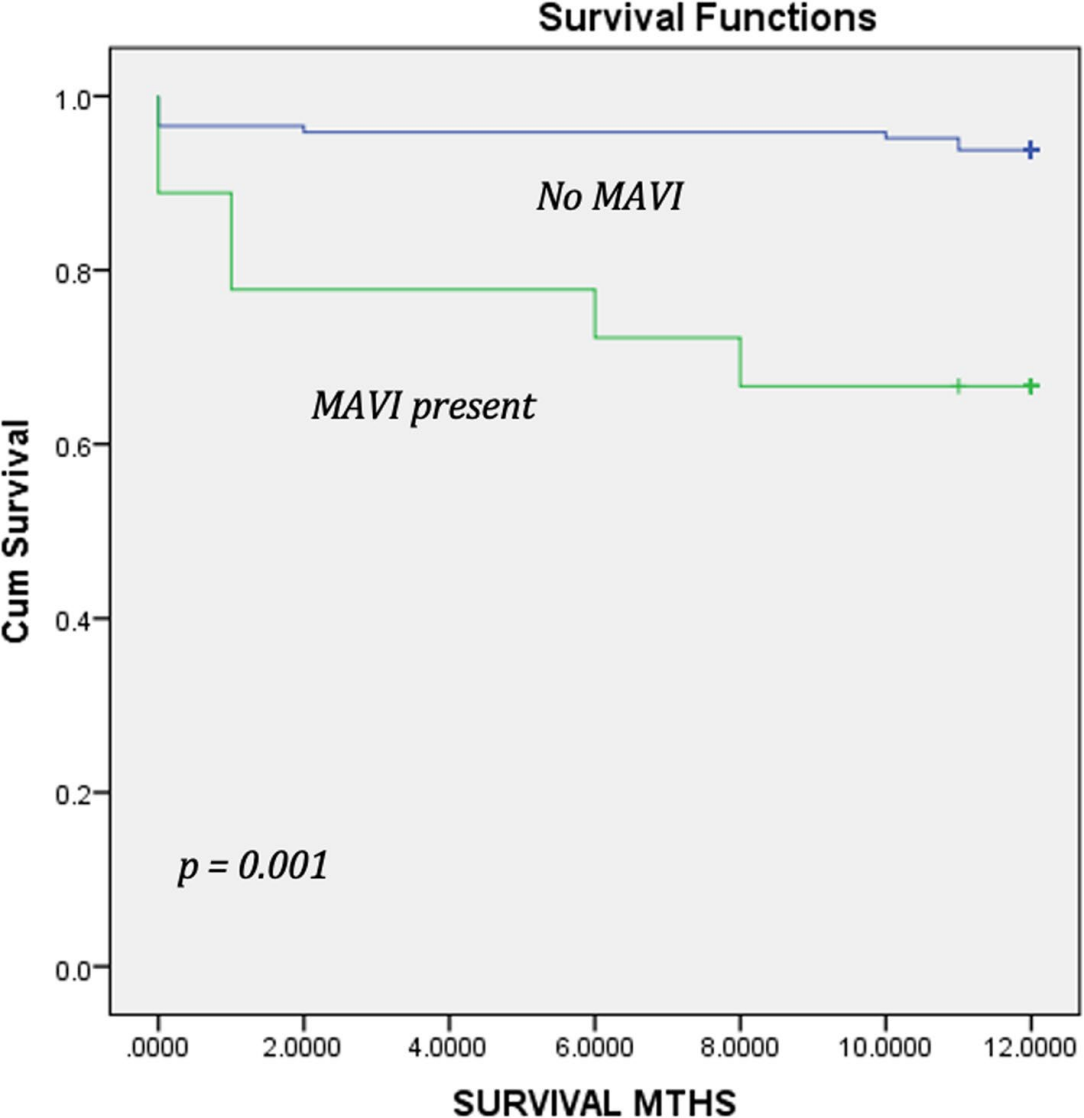
Kaplan–Meier survival curve for 1-year survival in patients with macrovascular invasion (MAVI), *p* = 0.001

**Fig. 4:**
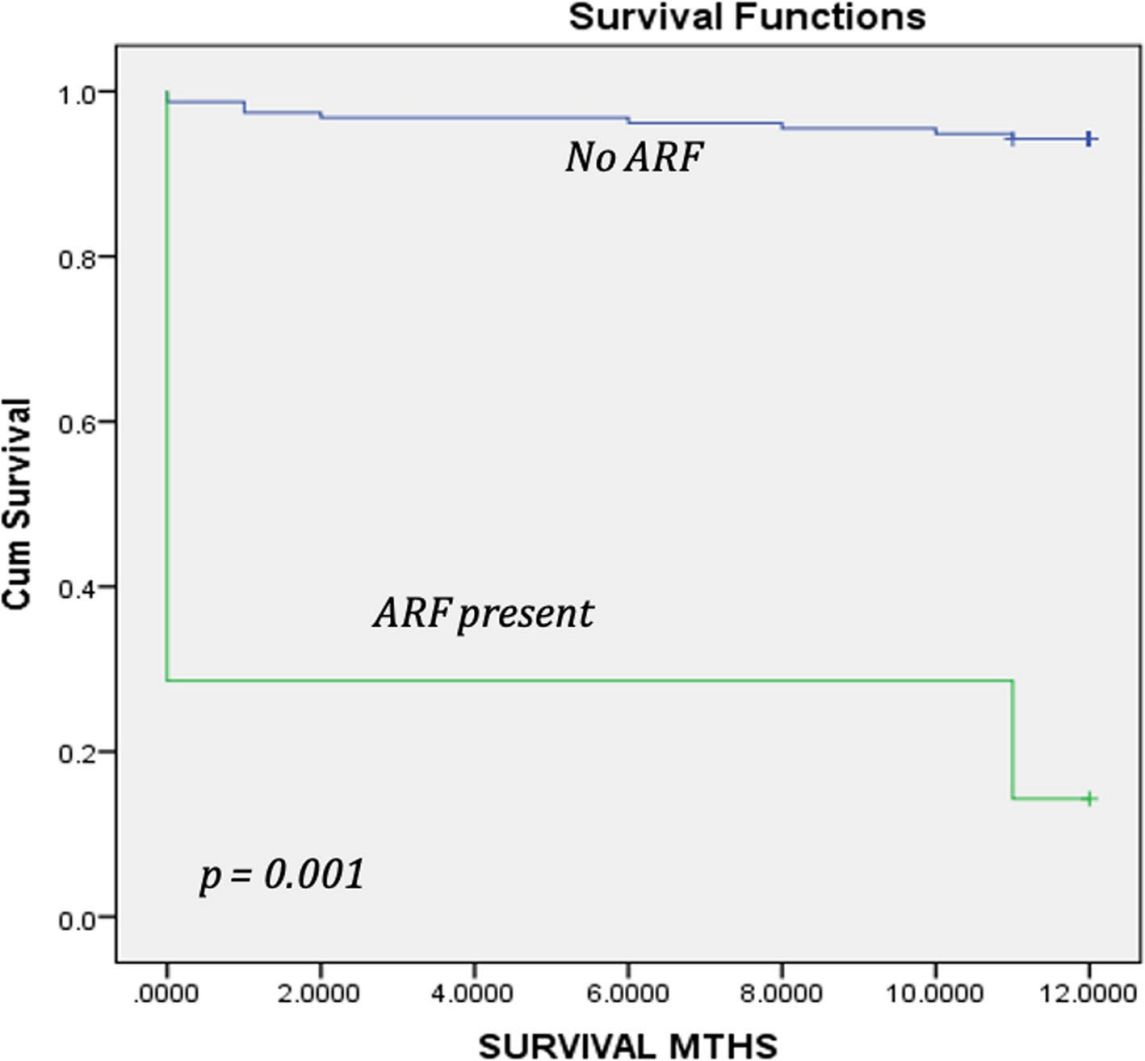
Kaplan–Meier survival curve for 1-year survival in patients with acute renal failure, *p* = 0.001

**Fig. 5:**
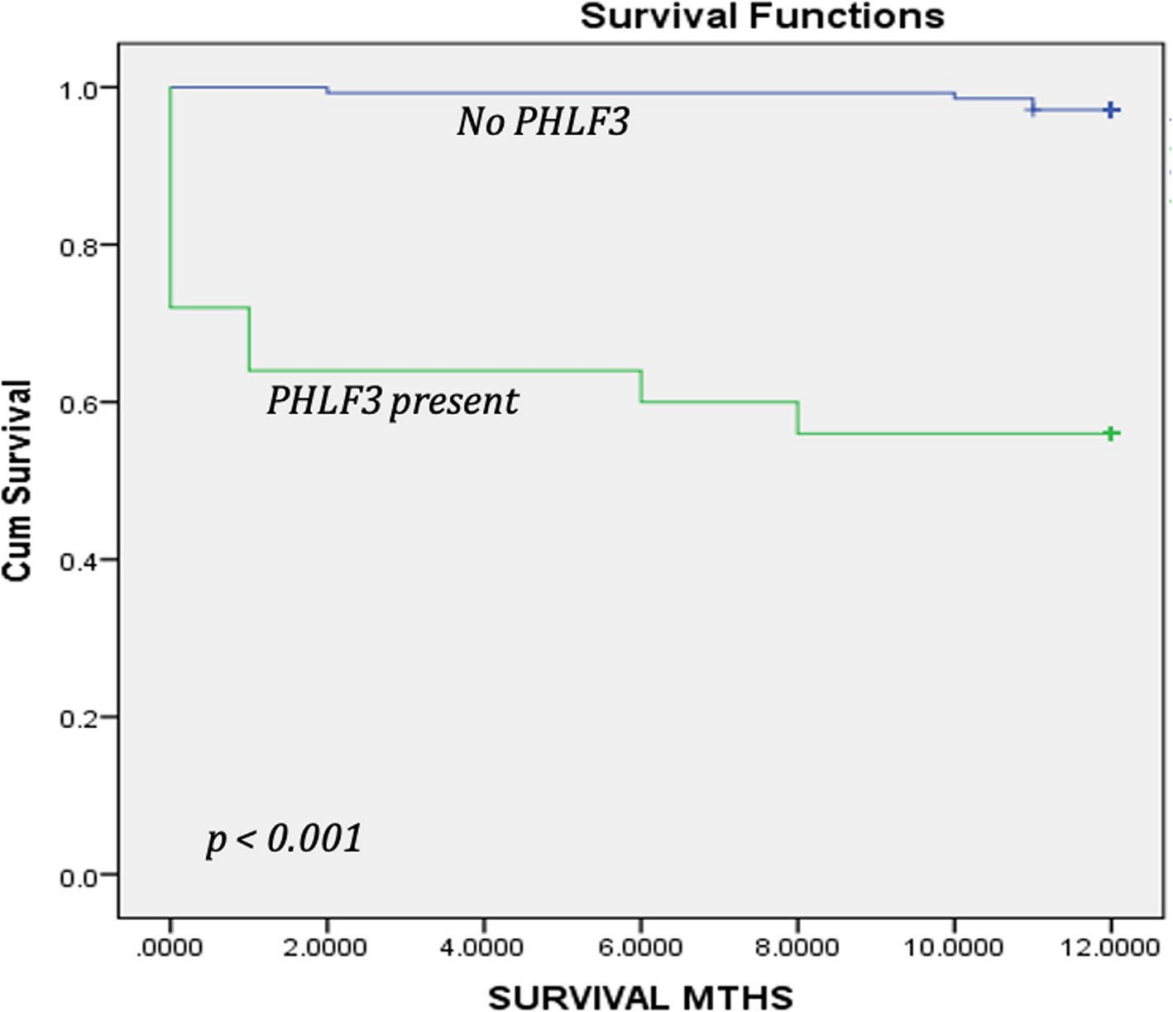
Kaplan–Meier survival curve for 1-year survival in patients with post-hepatectomy liver failure 3 (PHLF3), *p* < 0.001

Child-Pugh B or C, multinodularity, MAVI, ARF, and PHLF3 were independent risk factors for 1-year mortality with a decrease in the 1-year survival rate from 93.2 to 70.6% (*p* = 0.001) (shown in Fig. 1), 95.8 to 76.7% (*p* < 0.001) (shown in Fig. 2), 93.8 to 66.7% (*p* < 0.001) (shown in Fig. 3), 94.2 to 14.3% (*p* < 0.001) (shown in Fig. 4), and 97.1 to 56.0% (*p* < 0.001) (shown in Fig. 5), respectively.

## Discussion

Five independent factors were associated with mortality within 1 year in HCC patients undergoing HR with curative intent: Child-Pugh B/C, multinodularity, MAVI, ARF, and ISGLS PHLF criteria.

Cirrhotic patients have increased perioperative general risk for both elective and emergency surgical procedures [15, 16]. Our study showed that Child-Pugh stage B/C patients have a five-fold risk of 1-year mortality. Due to high perioperative risk, patients with Child-Pugh class C are not candidates for HR, and we included one patient with Child-Pugh class C. Cirrhosis is also associated with poor oncologic outcomes. A study by Kojiro Taura et al. [17] reported a 5-year survival rate of 81%, 54%, and 28% in non-cirrhotic, Child-Pugh class A, and Child-Pugh class B following HR, respectively. This is attributed to either a recurrence or development of metasynchronous HCC in cirrhotic liver. Few studies have evaluated the short-term prognosis of cirrhosis on post-hepatectomy patients. Chopinet et al. [18] reported acceptable short-term mortality and morbidity in cirrhotic patients (up to Child-Pugh class B, 7 points) and suggested that HR can be considered if patients have normal indocyanine green test and no portal hypertension. TACE, while conventionally considered a palliative procedure, has shown to improve survival outcomes and improve quality of life in patients with unresectable HCC [7]. Saviano et al. [8] reported no statistical difference in a 3-year overall survival in cirrhotic patients who underwent TACE–RFA combination versus HR in the presence of large solitary tumors. In a recent meta-analysis comparing TACE–RFA combination versus surgery, Gui et al. have concluded that TACE–RFA combination has similar long-term oncologic outcomes with the benefit of lower morbidity compared with surgery [7]. Child-Pugh class B/C patients should be carefully evaluated and preoperatively optimized to reduce the risk of PHLF and mortality, ensuring that HR remains safe [18], while considering TACE–RFA combination as an alternative therapy. TACE should be re-classified as a ‘curative adjunct’ rather than palliative in its intent.

Our study, consistent with a study by Moriguchi et al. [19], showed that multifocal HCC is a risk factor associated with 1-year mortality. The European Association for the Study of the Liver (EASL) recommended LT instead of HR as a first-line treatment for multinodular tumors [20]. They showed that the 5-year survival of LT in early HCC was comparable to those without malignancy (70%), and recurrence rates were less than 25%. However, due to the scarcity of donor organs, HR is still the first-line surgical management in patients with HCC. Based on our results, surgery may not be an ideal option for patients with Child-Pugh class B/C and multinodular HCC. Recently, some studies have shown good surgical outcomes in select patients with multinodular tumors and well-preserved liver function. Cheung et al. [21] compared HR with combined resection and ablation in multinodular HCC. They showed that the combined therapy had longer median survival (53 vs. 44.5 months). Knowing the number of tumors would allow the surgeon to discuss and offer treatment that includes a combination of ablation, chemoembolization, and surgery that is individualized to each patient. Patients with multinodular HCC should be carefully monitored for recurrence and offered enrolment into clinical trials. We did not study the pattern of 1-year mortality and hence unable to comment if this was associated with early HCC recurrence.

Our study showed that MAVI was associated with a five-fold risk for 1-year mortality and this is consistent with other reports [22]. MAVI of the hepatic or portal vein is part of the natural progression of HCC and is associated with a high recurrence rate even when treated with LT. Previously, portal vein thrombosis was considered a contraindication for LT, but techniques like thromboendovenectomy have emerged and redefined the role of LT and HR in patients with portal vein thrombosis [23]. Both portal and hepatic vein thrombosis are known to induce hepatic hypertrophy, which has the benefit in increasing the FLR, and hence MAVI is not an absolute contraindication of HR. In patients with MAVI, the median survival is less than 1-year, and BCLC recommends palliative Sorafenib treatment [24]. Further stratification based on MAVI location has shown that tumor with hepatic vein or vena cava invasion had a meager median survival of less than 5 months, with postoperative mortality of 28% [25]. This led to Mount Sinai Medical Centre no longer offering HR in patients with MAVI, as this result is worse than medical treatment or no therapy at all. Based on our experience and available literature, we would caution against HR in patients with MAVI and multifocal HCC as 1-year mortality risk is high, and the patient may enjoy survival advantage from TACE–RFA combination therapy [7]. Hepatic venous invasion and portal venous invasion may have a different biologic basis and need to be considered separately. A recent study by Levi Sandri et al. [26] achieved a recurrence-free survival of 39.1 months in four BCLC stage C patients with portal vein thrombosis by doing yttrium-90 radio-embolization before LT. Our study did not compare the outcome between location-stratified or perioperatively and intraoperatively known MAVI. Nevertheless, if MAVI is known on preoperative imaging, the patient should be counseled so that they can make informed decisions.

PHLF occurs in 4–19% of patients undergoing HR and is a severe complication that impacts mortality [27]. The variation in incidence arises due to the lack of a standardized definition. As part of postoperative factors, our study looked at three well-established definitions of PHLF. This variability of the definition is demonstrated in our study, where PHLF incidence was 3.7%, 4.3%, and 15.3% for PHLF1, 2, and 3, respectively. Only PHLF3 was statistically significant in the multivariate analysis of postoperative factors. In a study reporting 807 patients, Rahbari et al. [14] reported ISGLS criteria predicted mortality (HR 13.80; 95% *CI* 4.27–44.61, *p* < 0.01). Validation of PHLF3 as a postoperative 1-year mortality predictor is essential. PHLF is best prevented by optimization of preoperative factors [27] and incorporating strategies like portal vein embolization, two-staged hepatectomy^,^ and associating liver partition and portal vein ligation for staged hepatectomy (ALPPS) [28] to ensure satisfactory FLR. Integrating active monitoring of INR and bilirubin on postoperative day five can allow for early recognition of PHLF3, stratification of severity, and initiation of supportive organ therapy, and improving survival risk.

Postoperative ARF is a risk factor associated with 1-year mortality, which is consistent with a study by Saner et al. [29], that reported 73% of patients with post-hepatectomy ARF requiring dialysis died. In our study, 85% of ARF patients died, and seven patients (4.3%) experienced ARF. Lim et al. [30] suggested adequate volume expansion, use of diuretics or vasoactive drugs, and earlier postoperative renal replacement therapy for higher-risk patients.

Limitations of our study are retrospective, single-center study over a decade, and small sample size. Due to small sample and low event rate for mortality, the multivariate analysis showed wide confidence intervals. This impacts the precision of our study results. As there was only one patient with Child-Pugh class C, we have combined the data of Child-Pugh classes B and C. We did not categorize complications according to Clavien-Dindo classification and have reported each complication separately. We did not collect data related to the cause of mortality and if this was related or associated with the recurrence of HCC. We caution against generalizing the results as clinical profile, surgical technique, and technology and perioperative management would differ.

## Conclusions

In conclusion, our study showed that Child-Pugh B or C, multinodularity, and macrovascular invasion were independent predictors of mortality within the first year post curative hepatectomy, and postoperative renal or liver failure was associated with 1-year mortality. Knowing these risk factors can aid in clinical decision-making and the design of clinical trials.

## Data Availability

The data that support the findings of this study are available from the National Healthcare Group but restrictions apply to the availability of these data, which were used under license for the current study, and so are not publicly available. Data are however available from the authors upon reasonable request and with permission of the National Healthcare Group.

## Abbreviations

HR: Hepatic resection
HCC: Hepatocellular carcinoma
LT: Liver transplant
ALPPS: Associating liver partition and portal vein ligation for staged hepatectomy
DFS: Disease-free survival
OS: Overall survival
FLR: Future liver remnant
RFA: Radiofrequency ablation
TACE: Trans-arterial chemoembolization
PHLF: Post-hepatectomy liver failure
AFP: Alpha-fetoprotein
CT: Computed tomography
NLR: Neutrophil–lymphocyte ratio
CI: Confidence interval
MAVI: Macrovascular invasion
ARF: Acute renal failure
